# Immunologic Clearance of a BK Virus-associated Metastatic Renal Allograft Carcinoma

**DOI:** 10.1097/TP.0000000000003193

**Published:** 2021-01-26

**Authors:** Raphael P.H. Meier, Yannick D. Muller, Pierre-Yves Dietrich, Jean-Christophe Tille, Sergey Nikolaev, Ambra Sartori, Intidhar Labidi-Galy, Thomas Ernandez, Amandeep Kaur, Hans H. Hirsch, Thomas A. McKee, Christian Toso, Jean Villard, Thierry Berney

**Affiliations:** 1 Abdominal Transplant Surgery, Department of Surgery, Geneva University Hospital and University of Geneva Medical School, Geneva, Switzerland.; 2 Transplant Surgery, Department of Surgery, University of California San Francisco, San Francisco, CA.; 3 Immunology and Transplant Unit, Department Diagnostic, Geneva University Hospital and University of Geneva Medical School, Geneva, Switzerland.; 4 Department of Oncology, Geneva University Hospital and University of Geneva Medical School, Geneva, Switzerland.; 5 Diagnostic Department, Geneva University Hospital, and Department of Pathology and Immunology, University of Geneva Medical School, Geneva, Switzerland.; 6 Department of Genetic Medicine and Development, Geneva University Hospital and University of Geneva Medical School, Geneva, Switzerland.; 7 Division of Nephrology, Department of Medicine, University Hospital of Geneva, Geneva, Switzerland.; 8 Transplantation and Clinical Virology, Department Biomedicine, University of Basel, Basel, Switzerland.

## Abstract

**Background.:**

Metastatic carcinoma of a renal allograft is a rare but life threatening event with a difficult clinical management. Recent reports suggested a potential role of BK polyomavirus (BKPyV) in the development of urologic tract malignancies in kidney transplant recipients.

**Methods.:**

We investigated a kidney-pancreas female recipient with an history of BKPyV nephritis who developed a rapidly progressive and widely metastatic donor-derived renal carcinoma 9 years after transplantation.

**Results.:**

Histology and fluorescence in situ hybridization analysis revealed a donor-derived (XY tumor cells) collecting (Bellini) duct carcinoma. The presence of BKPyV oncogenic large tumor antigen was identified in large amount within the kidney tumor and the bowel metastases. Whole genome sequencing of the tumor confirmed multiple genome BKPyV integrations. The transplanted kidney was removed, immunosuppression was withdrawn, and recombinant interleukin-2 (IL-2) was administered for 3 months, inducing a complete tumor clearance, with no evidence of disease at 6-year follow-up. The immunological profiling during IL-2 therapy revealed the presence of donor-specific T cells and expanded cytokine-producing bright natural killer cells but no donor-specific antibodies. Finally, we found persistently elevated anti-BK virus IgG titers and a specific anti-BKPyV T cell response.

**Conclusions.:**

This investigation showed evidence for the potential oncogenic role of BKPyV in collecting duct carcinoma in renal allografts and demonstrated that immunosuppression withdrawal and IL-2 therapy can lead to an efficient antitumor cellular mediated rejection possibly via 3 distinct mechanisms including (1) host-versus-graft, (2) host-versus-tumor, and (3) anti-BKPyV responses.

## Abstract 2

Supplemental Digital Content is available in the text.

## INTRODUCTION

De novo renal carcinoma rarely occurs in kidney grafts, with an incidence rate ranging from 9 to 11 cases per 100 000 patient-years, and accounts for 0.3% of the tumors in transplanted patients.^1,2^ Treatment usually includes nephron-sparing surgery or radical graft nephrectomy. Among patients with a metastatic disease, a 50% fatal outcome is generally observed despite immunosuppression withdrawal, graft nephrectomy, cytotoxic therapy, immunotherapy, and radiotherapy.^1-3^ The management of de novo donor-derived metastatic tumors remains poorly defined, with only a few case reports in the literature.^4,5^ In the spectrum of renal carcinoma, collecting duct carcinoma or Bellini duct carcinoma represents a rare subset, different from the classic renal cell carcinoma, and accounting for 0.4% to 2% of cases.^6,7^ It is an aggressive tumor with 30% of patients having distant metastases at the time of diagnosis.^6^ Overall, metastatic collecting duct carcinoma is characterized by poor prognosis and a resistance to systemic chemotherapy with median survival ranging from 5 to 11 months.^8,9^ Over the past few years, it has been hypothesized that renal cell and collecting duct carcinomas could be linked to BK polyomavirus (BKPyV) in transplanted patients.^5,10-14^ BKPyV-associated nephropathy (PyVAN-B) complicates 8% (range, 1% to 15%) of kidney transplant recipients causing premature graft loss in 10% to 50% of cases.^15^

We report the successful management of a kidney-pancreas recipient who developed de novo multimetastatic donor-derived collecting duct carcinoma. We found the presence of large tumor antigen (LTag) in the primary tumor and metastases and multiple BKPyV integrations into the genome of tumor cells. The patient developed a strong and sustained host-versus-graft, -tumor, and -BKPyV cellular response, which was enhanced by immunosuppression withdrawal and interleukin-2 (IL-2) therapy. We detected a peripheral expansion of donor-specific CD8^+^ T cells and activated NKp30^+^ NKp46^+^ natural killer (NK) cells and a specific anti-BKPyV T cell response.

### Case Presentation

**FIGURE 1.**
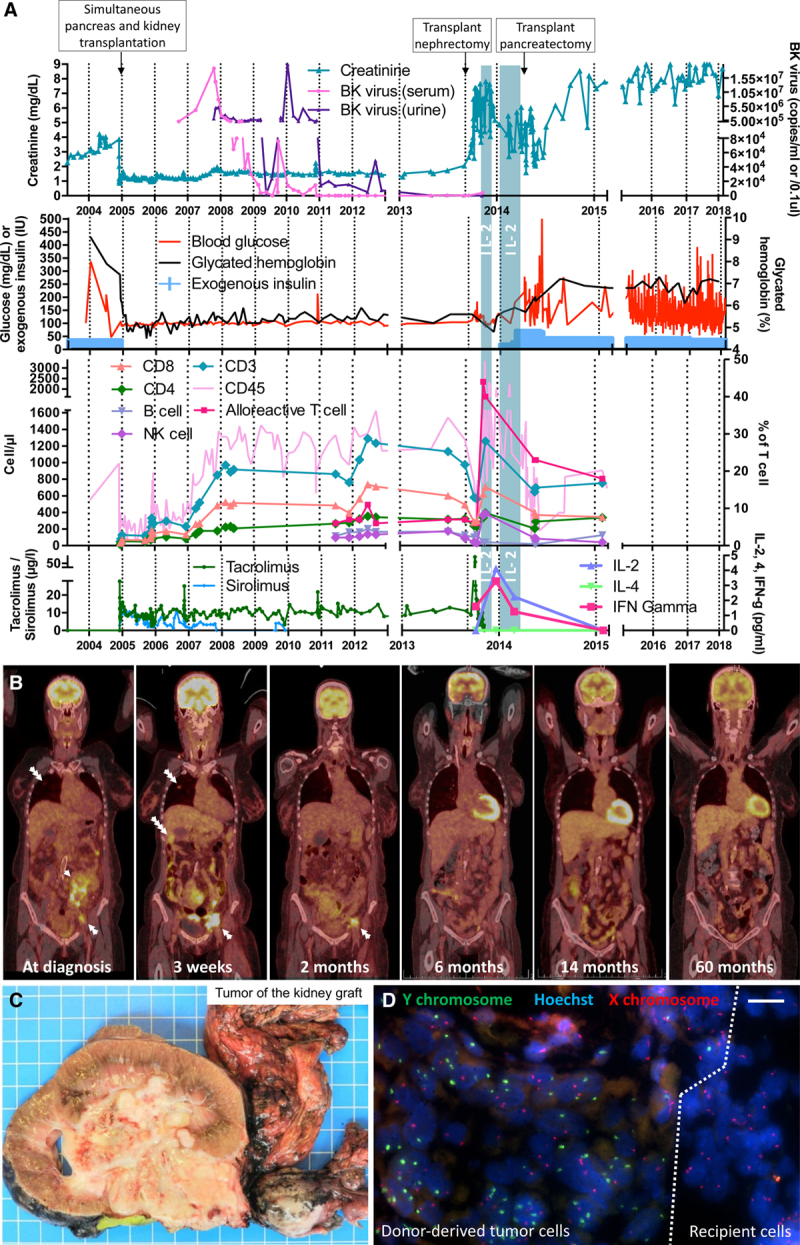
Clinical, biological, radiological, and pathological evolution of the kidney-pancreas and the collecting duct carcinoma. A, Chart depicting the 15-y evolution of patient’s renal and endocrine pancreas function. BK virus copies per mL (blood) or per 0.1 μL (urine) are represented. The count of the different peripheral blood mononuclear cell populations was assessed over time. Interleukin (IL)–2 treatment course is shown as vertical blue bars and serum IL-2, -4, and interferon-γ (IFN-γ) levels preceding and following IL-2 treatment are represented. Immunosuppression regimen including tacrolimus and sirolimus trough levels are represented. B, PET/CT scans showing a metastatic collecting duct carcinoma at diagnosis (3 d before transplant nephrectomy) and 3 wk after surgery. Two-, 6-, 14-, and 60-mo follow-up imaging showing disappearance of the tumor. Single white arrow, kidney tumor; double white arrow, iliac metastatic lymph nodes; triple white arrow, lung metastasis; and quadruple white arrow, liver metastasis. C, Macroscopic view showing the explanted kidney graft invaded by the collecting duct carcinoma (1 blue square: 1 cm). D, fluorescence in situ hybridization (FISH) analysis of a kidney cryosection showing Y chromosome presence (in green) at the junction between tumor tissue and surrounding recipient parenchyma; tumor cells including multiple Y chromosome copies due to the high mitosis rate (scale bar: 50 µm). IU, international unit; NK, natural killer; PET/CT, positron emission tomography/computed tomography.

A 41-year-old nonsmoking woman with type I diabetes since the age of 10 received a combined kidney-pancreas allograft from a 20-year-old male donor who died of cerebral trauma (neurologically determined death). The posttransplant period was uneventful; euglycemia was rapidly achieved, insulin treatment was discontinued, and the patient was discharged with creatinine levels of 1.37 mg/dL (Figure 1A). Induction therapy consisted in antithymocyte globulin (d 0 to d 3) and methylprednisolone (steroids were tapered down then withdrawn over a period of 6 wk), and maintenance in a tacrolimus-sirolimus combination. Drug levels were monitored daily in the immediate postoperative period (until discharge), once a week in the month following discharge, and once a month thereafter. Tacrolimus goals were 8–12 ng/mL for the first 6 months and then 7–10 ng/mL (trough levels are shown in Figure 1A). She received an initial 6-month course of valganciclovir because of donor+/recipient– cytomegalovirus (CMV) serologies. One-year posttransplantation, she developed asymptomatic CMV primoinfection, successfully treated with intravenous ganciclovir and anti-CMV antibodies. Two years’ posttransplant, creatinine levels increased from 1.20 to 1.82 mg/dL. A renal biopsy demonstrated a PyVAN-B. A positive BKPyV-DNAemia up to 1.4 × 10^7^ copies/mL was also detected (Figure 1A). BKPyV-DNAemia returned to undetectable values 1 year after switching to tacrolimus monotherapy. At that time, sirolimus reintroduction was attempted but led to recurrence of BKPyV-DNAemia. The 2-year control biopsy showed chronic tubulointerstitial nephritis with undetectable LTag staining.

Nine-year posttransplant, a routine ultrasound (performed yearly at our institution) revealed the presence of a cortical cyst within the transplanted kidney. Two successive MRIs confirmed the presence of a Bosniak IIF cyst extending into of the hilum that enlarged from 2 to 9 cm within 6 months. A positron emission tomography/ computed tomography (PET/CT) scan showed a renal mass (maximum standardized uptake value of 13.0) and multiple hypermetabolic lesions in the peritoneal cavity, mesentery, iliac lymph nodes, and lungs suggesting a metastatic kidney malignancy (Figure 1B). Graft nephrectomy was performed and biopsies of small bowel/lymph nodes/greater omentum metastases were taken. Histopathology revealed a metastatic, donor-derived, collecting duct carcinoma, stage pT3a pN1 pM1 (Figure 1C). The donor being male, fluorescence in situ hybridization analysis was performed, showing a XY karyotype, thus confirming donor’s origin of the tumor (Figure 1D). Three weeks after surgery, a new PET/CT scan showed a rapid progression of the disease with an increase in size of iliac, peritoneal and mesenteric lesions, and appearance of new hepatic and thoracic lesions (Figure 1B).

In the absence of efficient chemotherapy options, a host-versus-graft/tumor response strategy was pursued. To that end, immunosuppression was withdrawn and IL-2 was administered at 6 million IU subcutaneously qd (5d/7) for 3 months. IL-2 side effects included fever, shivering, redness at the sites of injection, transient bilateral pleural effusion, and a 20 kg weight loss. During the treatment course, the patient was taken off IL-2 during 3 weeks due to the severity of the side effects. She returned to dialysis and insulin therapy was reintroduced 2 months after nephrectomy (Figure 1A). Six months after the cessation of immunosuppression, she presented with a spontaneous rupture of pseudoaneurysm of the pancreas arterial anastomosis, requiring emergency surgical exploration for hemostasis. Of note, only an atrophic pancreatic graft remnant could be identified during the surgery, this speaking in favor of a complete immune rejection. The 3- and 6-month PET/CT scans showed regression of peritoneal, liver, and lung metastatic lesions and all hypermetabolic lesions disappeared at the 14-month PET/CT control and remained so at 60 months follow-up (Figure 1B). After 6-year follow-up, the patient is alive, on hemodialysis and insulin therapy, without evidence of cancer. Of note, none of the 3 other recipients from the same donor (heart, right kidney, and liver) developed malignancies.

### Tumor Phenotype

A 4.5-fold increase in tumor size was observed within 6 months. Histopathology of the explanted kidney confirmed the diagnosis of a collecting duct carcinoma with areas of tumor necrosis, multiple lymphovascular invasions, and invasion of surrounding structures (ovary, muscle). Tumor cells stained positive for total keratin, cytokeratin 7, cytokeratin 20, paired box gene 8, and integrase interactor 1; and negative for carbonic anhydrase 9 (renal cell carcinoma), p63, Wilms' tumor 1, cytokeratin (CK)5.6, CK14, CK903, octamer-binding transcription factor 4, and HLA-DR (**Figure S1**, **SDC**, http://links.lww.com/TP/B888). No B or T lymphocyte infiltration or C4d deposition was seen in the tumor (**Figure S2**, **SDC**, http://links.lww.com/TP/B888). A mild macrophage (CD68^+^ cells) infiltrate was observed.

### BK Polyomavirus

**FIGURE 2.**
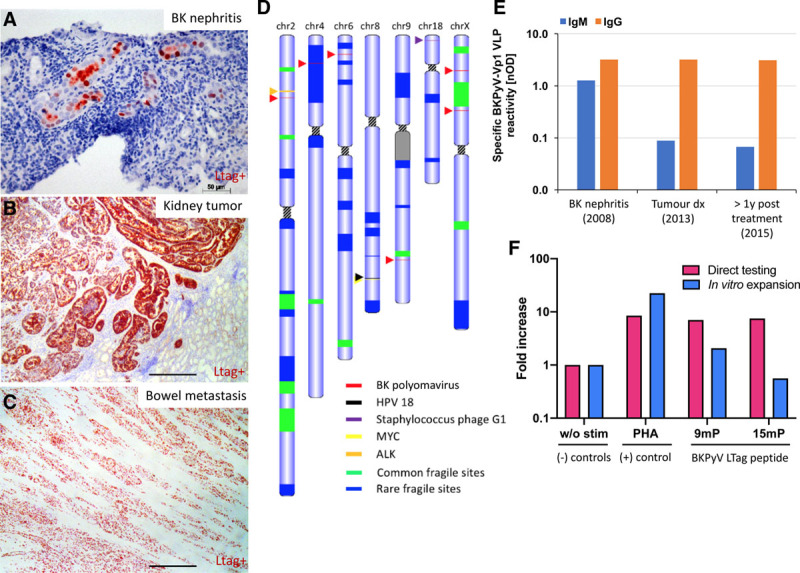
BK polyomavirus (BKPyV) integration in the collecting duct carcinoma and elicited humoral and cellular immune response. A, Large tumor antigen (LTag) staining (in red) indicating the presence of the BK virus in the kidney during the BK nephritis (before the tumor development), (B) in the kidney tumor, and (C) in the small bowel metastasis. Scale bar: 400 µm unless indicated. D, Tumor karyotype showing chromosomes with any known viruses’ integration sites and close by tumor suppressor/oncogene presence. E, Specific anti-BK virus IgM and IgG titers (against viral capsid protein 1 virus-like particles) during BK nephritis, at tumor diagnosis and 14 mo after tumor removal (labeled >1 y). F, BKPyV-specific T cell responses were quantified using interferon-γ (IFN-γ) release by enzyme-linked immunospot (ELISpot) assay using peripheral blood mononuclear cell (PBMC) collected when the patient was in remission (5 y after diagnosis). PBMCs were either stimulated directly (pink bars) or stimulated with 15mP covering the BKPyV-LTag for a 2 wk expansion in vitro before retesting (blue bars) for IFN-γ release by ELISpot. Negative control: without peptides (w/o stim); positive control: phytohemagglutinin-L (PHA-L); BKPyV peptide pools: 97 immunodominant 9mers of BKPyV EVGR (9mP) and 180 15mers overlapping by 11 amino acids spanning the entire BKPyV LTag (15mP). The results are expressed as fold increase of IFN-γ spot-forming units (SFUs) relative to negative control. PBMC response was performed in technical duplicate (direct testing) and triplicate (after in vitro expansion). ALK, anaplastic lymphoma kinase; HPV-18, human papillomavirus 18; MYC: MYC proto-oncogene; nOD, Net optical density.

Immunohistochemistry was positive for simian virus 40 (SV40)-LTag and the diagnosis of PyVAN-B was made. In addition, LTag-expression was demonstrated in the primary tumor and metastases (Figure 2A–C). Staining for the BKPyV major capsid protein Vp1 was negative in the tumor tissues. A whole genome sequencing of the tumor found multiples BKPyV integration sites in chromosome 2, 4, 6, 9, and X (Figure 2D). On chromosome 9, 11 multiple viral reads were clustered in <800 bp length. We incidentally found sequences of the human papillomavirus 18 and Staphylococcus phage G1 integrations on chromosome 8 and 18, respectively. High BKPyV-specific IgG titers were detected as well as transiently elevated IgM during BKPyV nephropathy (Figure 2E). Five years after diagnosis, the patient showed BKPyV-specific CD8^+^ T cell responses after direct stimulation of peripheral blood mononuclear cells (PBMCs) using 97 immunodominant 9mer peptide pool derived from the early viral gene region (60 interferon [IFN]–γ spot-forming units/106 PBMCs) (Figure 2F). The response was also detected following in vitro expansion and stimulation with 97 immunodominant 9mer peptide pool (253 spot-forming units/106 PBMCs). These data suggest that BKPyV incorporated its genome in donor-derived renal cells despite humoral immunity. The omnipresence of BKPyV in the tumor is in line with the notion that viral DNA integration in the tumor cells led to persistent upregulation of early gene proteins in the absence of chronic active replication.

### Cellular-mediated Antitumor Response

**FIGURE 3.**
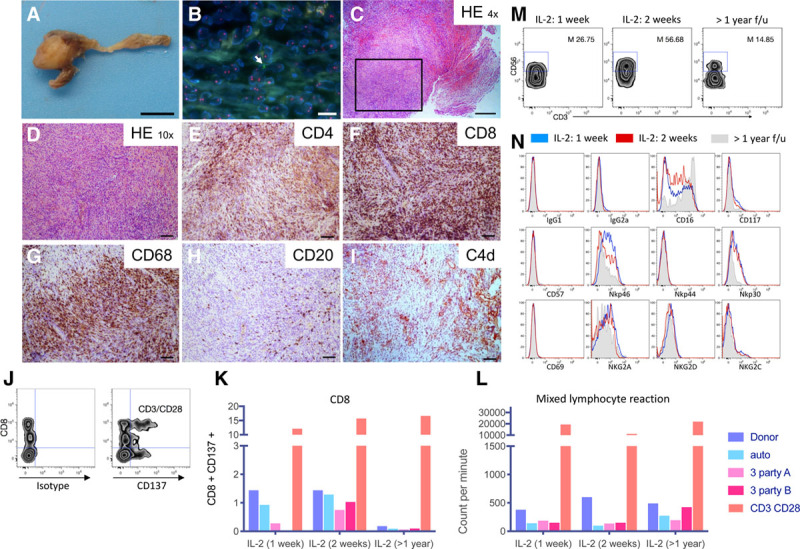
Cellular-mediated antitumor response. Macroscopic (A) and microscopic (B–I) view of the pancreatic remnant specimen retrieved around pancreatic Y graft artery 6 mo after the transplant nephrectomy. Macroscopic view scale bar: 1 cm. B, Fluorescence in situ hybridization (FISH) analysis showing rare donor cells positive for Y chromosome (arrow) and X chromosome in red. Scale bar: 10 µm. C and D, Hematoxylin and eosin staining. Scale bar: 400 and 100 µm, respectively. E–I, Immunohistochemistry staining for (E) CD4, (F) CD8, (G), CD68, (H) CD20, and (I) C4d. Scale bars: 100 µm. J, Gating strategy showing CD137 and CD8^+^ T cell analysis gated on patient’s live peripheral blood mononuclear cells cocultured with irradiated PE-labeled autologous cells, donor cells, two-third party stimulator cells or with CD3/CD28 beads for 36 h. K, CD8^+^ T cells quantification at 1 wk, 2 wk, and 14 mo after interleukin-2 (IL-2) treatment initiation (labeled >1 y). L, Mixed lymphocyte reaction of patient peripheral blood mononuclear cells cocultured with irradiated autologous cells, donor cells, third party stimulators or with CD3/CD28 beads at 1 or 2 wk after IL-2 treatment initiation and 14 mo after IL-2 treatment (performed in technical triplicates). After 6 d of culture, the proliferation was measured by thymidine incorporation and is expressed as counts per min. M, Gating strategy to define the percentage of CD3^–^ CD56^+^ bright natural killer (NK) cells in the patient’s NK cell population (defined as CD3^–^ CD56^+^) at 1 or 2 wk after IL-2 treatment initiation and 14 mo after IL-2 treatment (M: mean). N, Immunophenotypic analysis (IgG1 and IgG2a controls, CD16, CD57, Nkp46, NKp44, NKp30, CD69, NKG2a, NKG2d, NKg2C) of CD3^–^ CD56^+^ NK cells in recipient’s peripheral blood mononuclear cells at different time points (1 wk IL-2 [gray], 2 wk IL-2 [black], or 14 mo after IL-2 treatment [shaded gray]). 7AAD^+^ positive dead cells were excluded of the analysis. f/u, follow-up; HE, hematoxylin eosin; NKG, NK cell group; PE, phycoerythrin.

We characterized the immune infiltrate of the pancreatic remnant specimen taken at the time of pseudoaneurysm rupture, that is, 6 months after immunosuppression withdrawal (Figure 3A). Fluorescence in situ hybridization analysis revealed some rare positive XY cells (donor) within the granulation tissue suggesting that it may represent the rejected pancreatic graft remnant (Figure 3B). Histology and immunohistochemistry analysis showed a cellular infiltration of CD8^+^, CD4^+^ T cells, and CD68^+^ macrophages (Figure 3C–F). C4d deposition, IgG, and IgM deposition were negative with only a few CD20^+^ B cells present speaking against an active humoral response (Figure 3H and I). Class-I donor-specific antibodies were mostly negative except for 2 positive testing in 2010 of 2012 (before tumor diagnosis) and remained negative. Class-II donor-specific antibodies were always negative. Mixed lymphocyte reaction demonstrated donor-specific proliferation of PBMCs as well as activation of CD8^+^ T cells 1 and 2 weeks after IL-2 treatment initiation compared to third party donors (Figure 3J–L). At the 14-month follow-up testing, the activation of CD8^+^ T cells was not detectable (Figure 3K), while the alloreactive proliferation is still detectable, although to a lesser extent (Figure 3L).

### Peripheral Activation of T Cells and Natural Killer Cells

During IL-2 therapy, we observed an increase in absolute numbers of CD4^+^, CD8^+^, and NK cells up to 1.7-, 2.3-, and 11.4-fold, respectively (Figure 1A). HLA-DR expression on T cells increased from 6% to 44% as early as 1 week after IL-2 treatment. Flow cytometry analysis showed that the percentage of cytokine-producing bright NK cells increased up to 56% during IL-2 therapy (Figure 1A). NK cells upregulated the natural cytotoxic receptors NKp30 and NKp46, both associated with antitumor response (Figure 3M and N).

## DISCUSSION

We describe the treatment and remission of a metastatic donor-derived BKPyV-associated collecting duct carcinoma with IL-2-based immunotherapy and immunosuppression withdrawal.

The treatment of metastatic collecting duct carcinoma is heterogeneous and various and largely unsuccessful options have been described, including gemcitabine, cisplatin, methotrexate, vinblastine, adriamycin, paclitaxel, carboplatin, IFN-α, IFN-γ, and IL-2.^16^ Immune checkpoint inhibitors were recently added to this list.^17^ Among these, IL-2 was 1 of the first anticancer immunotherapy and was approved for the treatment of metastatic renal cell carcinoma and metastatic melanoma in the 90’s by the Food and Drug Administration.^18^ Historically, salvage therapies included IL-2^17,19^ and allogeneic bone marrow transplantation^20^ which allowed a complete response in <10% of the cases. However, IL-2 lost favor due to its toxic side effect profile. Currently, the role of IL-2 is reemerging as first-line immunotherapy^18^ in combination with cell-based immunotherapy, peptide vaccines, and other chemotherapeutic agents. Studies using IL-2 in combination with anti-programmed cell death 1, entinostat, or tumor-infiltrating lymphocytes are currently underway, recruiting, and treating patients (eg, NCT02926053, NCT01884961, NCT03260504, NCT02306954, NCT03474497, and NCT03991130). The clinical decision, to administer IL-2, in the context of a particularly aggressive tumor type, was taken, because immunosuppression withdrawal alone cannot prevent tumor recurrence in a significant proportion of de novo^1,11^ and transmitted^21^ donor-derived tumors. The 3 potential mechanisms by which the patient cleared the tumor include (1) alloimmune rejection,^22^ (2) antitumor response, and (3) antiviral response. Given the pleiotropic effects of IL-2 on the immune system, it may have promoted all 3 types of responses. It was not possible to determine the exact importance of each of these factors individually, and we cannot exclude that immunosuppression alone could have led to the same favorable outcome. One can speculate that a multiple target strategy against the neoplasm gave our patient the best chance to achieve complete remission. Another aspect potentially involved in the immunologic clearance of the tumor is the long-lasting adjuvant role of the remaining pancreas allograft. The polyclonal response against multiple donor epitopes present in the pancreatic graft left in place may have helped tumor rejection. Such strategy has also recently been employed in a metastatic donor-derived renal cancer patient in whom transplantectomy was intentionally delayed to elicit a broad alloimmune antitumor response.^4^

An important observation made in the present study is the compelling evidence that the development of the tumor was linked to BKPyV, namely, BKPyV genome integration and viral protein expression into/by the tumor. Polyomaviruses, including BKPyV, human JC polyomavirus, and SV40, encode 2 viral oncogenes, the large and small T antigen.^23^ These viral oncoproteins can inactivate tumor suppressor genes p53 and pRb and induce human cell transformation into cancer.^23^ It was suggested that the integration of the BKPyV results in alterations in viral gene expression, including a disruption of viral protein 1 protein expression and sustained expression of large T-antigen, accompanied with a shutdown of viral replication and deletions in the noncoding control region.^24^ Of note, the breakpoint sequences where the BK virus integrates into the genome and the nature of genes affected seems not to play a predominant role in oncogenesis.^25^ Overall, our observations are consistent with a role of BKPyV in the development of urologic tract malignancies in transplanted patients and further supports growing evidence.^5,10-13,25^ In our view, the lesson from this case is the importance of BKPyV screening and we would advocate a higher index of suspicion for urinary tract malignancies in patients with a former BKPyV infection.^26^ While our data and those of others highly suggest an oncogenic role of the BKPyV, we think that, paradoxically, BKPyV may also participate to tumor clearance be it due to lytic replication or due to the HLA-class I presentation of viral antigens,^23^ as is the case for human endogenous retrovirus.^27^ This mechanism, along with host-versus-graft and host-versus-tumor responses, can be stimulated by the administration of IL-2.

In conclusion, this case showed evidence for the potential oncogenic role of BKPyV in collecting duct carcinoma and demonstrated that immunosuppression withdrawal and IL-2 therapy lead to an efficient antitumor cellular mediated rejection with complete remission of a widely metastatic solid tumor.

## ACKNOWLEDGMENTS

We would like to thank the patient for allowing us to report her case to the medical community. As per our institutional guidelines for single-case report, this study was exempt from approval from our ethics board. We thank Florence Bettens for her help with the mixed lymphocyte reaction protocol. We thank Klaudia Nägele for advice on the interpretation of viral sequences detected by next generation sequencing data. We would like also to thank Solange Moll for her guidance and interpretation of the kidney histology. We thank Jörg Seebach for sharing reagents. Finally, we would like to thank all the medical doctors, nurses, pharmacists, and laboratory technicians who were involved in the management of the patient, particularly Nicolas De Picciotto. Detailed methods and supplementary figures are provided in the Supplemental Appendix.

## Supplementary Material


