# Supplementary material for: Immunologic Clearance of a BK Virus-associated Metastatic Renal Allograft Carcinoma
Source: Transplantation. 2021 Jan 26;105(2):423–9. doi: 10.1097/TP.0000000000003193 (PMC7837753; doi:10.1097/TP.0000000000003193)
Supplement: Supplementary file 1 [file tp-105-423-s001.pdf]

Supplemental appendix to:

Complete Response to IL-2 in a BK-associated Metastatic Kidney Graft Carcinoma

Raphael P.H. Meier, MD, PhD,[1,2] Yannick D. Müller, MD, PhD,[2,3] Pierre-Yves Dietrich, MD,[4] Jean-Christophe Tille, MD, PhD,[5] Sergey Nikolaev, PhD,[6] Ambra Sartori, MSc,[6] Intidhar Labidi-Galy, MD, PhD,[4] Thomas Ernandez, MD,[7] Amandeep Kaur, MSc,[8] Hans H. Hirsch, MD,[8] Thomas A. McKee, MD,[5] Christian Toso, MD, PhD,[1] Jean Villard, MD,[3,7] and Thierry Berney, MD, MSc[1]

1. Visceral and Transplant Surgery, Department of Surgery, Geneva University Hospital and University of Geneva Medical School, Geneva, Switzerland

2. Transplant Surgery, University of California San Francisco, San Francisco, CA, United States.

3. Immunology and Transplant Unit, Division of Immunology and Allergy, Geneva University Hospital and University of Geneva Medical School, Geneva, Switzerland

4. Center of Oncology, Geneva University Hospital and University of Geneva Medical School, Geneva, Switzerland

5. Diagnostic Department, Geneva University Hospital, and Department of Pathology and Immunology, University of Geneva Medical School, Geneva, Switzerland

6. Department of Genetic Medicine and Development, Geneva University Hospital and University of Geneva Medical School, Geneva, Switzerland.

7. Division of Nephrology, Department of Specialties, University Hospital of Geneva, Geneva, Switzerland

8. Transplantation & Clinical Virology, Department Biomedicine, University of Basel, Petersplatz 10; CH-4009 Basel, Switzerland

Table of contents

Supplementary Methods..3

Immunohistology 3

Flow cytometry 3

Mixed Lymphocyte reaction...3

Measurement of serum glucose, creatinine, glycated hemoglobin, FK506, and BK virus copies 4

Genome sequencing and viral DNA integration detection...4

Measurement of T cell responses to BK Polyomavirus 5

Supplementary figure and legend..6

References 8

Supplementary Methods

Immunohistology

Formalin-fixed tissues were embedded in paraffin, and 5 μm thickness sections were immunostained with antibodies antitotal keratin, cytokeratine (CK) 7, 20, PAX8, INI1, CA9, p63, WT1, CK5.6, CK14, CK903, OCT4, HLA-DR, CD4, CD8, CD20, CD68, C4d, CD56 and CD31). Y chromosome was detected in tumor tissue by fluorescence in situ hybridization (FISH).

Flow cytometry

Peripheral blood mononuclear cells (PBMC) were collected and frozen in DMSO while the patient was under IL-2 therapy (1 and 2 weeks after the first IL-2 injection) and 14 months after nephrectomy (patient in remission). PBMC were stained with IgG1, CD3, CD56, CD16, CD117, CD57, NKG2A, NKG2D, CD69, NKp46, NKp44, NKp30 and NKG2C. Cells were gated on living cells after exclusion of 7AAD positive cells. Cells were acquired on Attune Cytometer (Thermo Fisher Scientific, Waltham, MA, USA). Luminex (Austin, TX, USA) assay was used to detect antidonor and anti-islet of Langerhans antibodies.

Mixed Lymphocyte reaction

Frozen PBMC from the patient (HLA A1, A23, B8, B40, DR3 DR4) were stimulated with irradiated (4000 Gy) autologous PBMC (negative control), frozen splenocytes from the donor (HLA A3, A2, B7, B44, DR4 DR11), 2 third party PBMC (HLA A3, A2, B39 B57, DR1 DR7 = third party 1) (randomized PBMC = third party 2) and alternatively with CD3/CD8 Dynabeads (life technologies, 1:1 cells/beads ratio, positive control). 0.1 x10^6 patient responder cells' (rIL-2 one week, rIL-2 two weeks,

14 months) were mixed with 0.1 x10^6 stimulator irradiated (40Gy) cells (autologous, donor cells, third party 1 and 2) and 0.05 x10^6 responding cells were stimulated with 1:1 CD3 CD28 beads as positive control. On day 6, cells were pulsed with 1µCi^3[H] of thymidine for 18 hours and harvested. Results are expressed as counts per minute (cpm). Regarding CD8/CD137 analysis, experiments were performed as previously described.[1] Briefly, PBMC were cocultured for 36h with cocultured with irradiated PE-labeled autologous cells, donor cells, 2 third party stimulator cells or with anti-CD3/ CD28 beads. CD137 analysis gated on CD8 T cells was performed by flow cytometry.

Measurement of serum glucose, creatinine, glycated hemoglobin, FK506, and BK virus copies

Serum glucose, creatinine, and glycated hemoglobin were measured using UniCel DxC 800 Synchron Clinical Systems (Beckman Coulter, Brea, CA). FK506 levels were measured by ELISA. Serum and urine BK virus copies were quantified by RT-PCR.

Genome sequencing and viral DNA integration detection

The collecting duct tumor DNA was extracted from a frozen section biopsy using Qiagen DNeasy Mini kit (Qiagen, San Diego, CA, USA) according to the manufacturer's instructions. DNA quality control and tumor genotype was obtained using Illumina HiSeq X Whole Genome Sequencer (Illumina, San Diego, CA, USA). 1 473 044 500 reads were produced, and total read bases were 222.4G bp. The GC content was 43.72%, Q20 was 88.27% and Q30 was 77.17%. Virus integration detection was performed by paired end reads using Vy-PER software (Institute of Clinical Molecular Biology, Kiel University University Hospital, Kiel, Germany). We

used the GRCh37 assembly (Ensembl, http://www.ensembl.org) as a reference genome. Karyotype graph was designed using the institute for systems biology online software (http://db.systemsbiology.net/gestalt/cgi-pub/genomeMapBlocks.pl).

Measurement of T cell responses to BK Polyomavirus

BK Polyomavirus (BKPyV)-specific T cell responses were quantified using interferon-γ (IFN- γ) release by enzyme-linked immunospot (ELISpot) assay using peripheral blood mononuclear cells (PBMC) collected 5 years after initial diagnosis. PBMCs (250 000 PBMCs per microtiter well) were either stimulated directly or stimulated with 15mP covering the BKPyV-LTag for a 2-week expansion in vitro before retesting for IFN- γ release by ELISpot as previously described.[2,3] Negative control without peptides and positive control with phytohemagglutinin-L (2mg/ mL; Hoffmann-La Roche Ltd., Basel, Switzerland) were performed using 50 000 cells per well. BKPyV peptide pools included 9mP (97 immunodominant 9mers of BKPyV EVGR) and 15mP (180 15mers overlapping by 11 amino acids spanning the entire BKPyV LTag) at 1 lg/ mL (Eurogentec, Belgium). Spot-forming units were enumerated by an ImmunoSpot analyzer (CTL Europe GmbH, Bonn, Germany).

Supplementary figures and legends

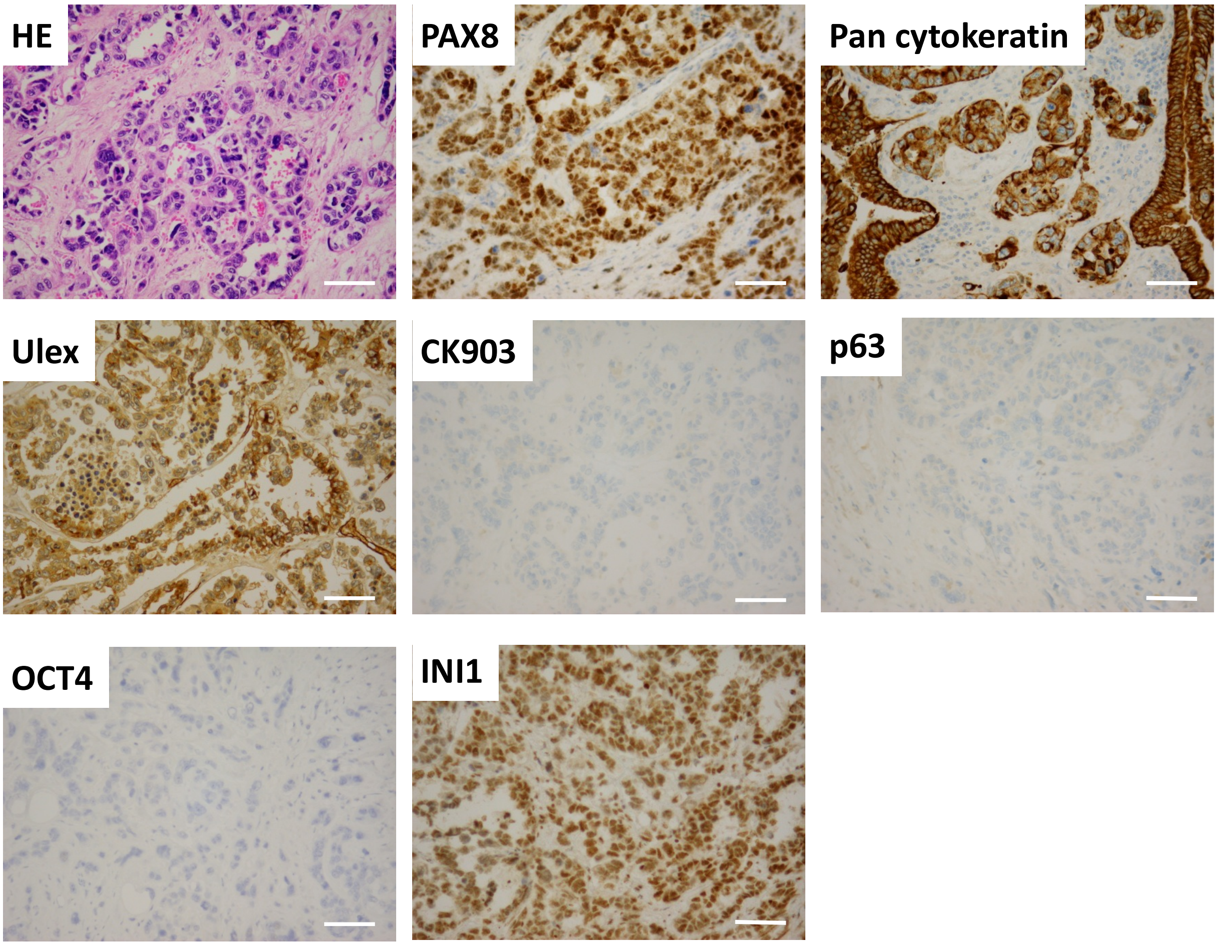

Figure S1: Histological section of the kidney showing typical positive and negative makers of the collecting duct carcinoma. Scale bar: 6 µm.

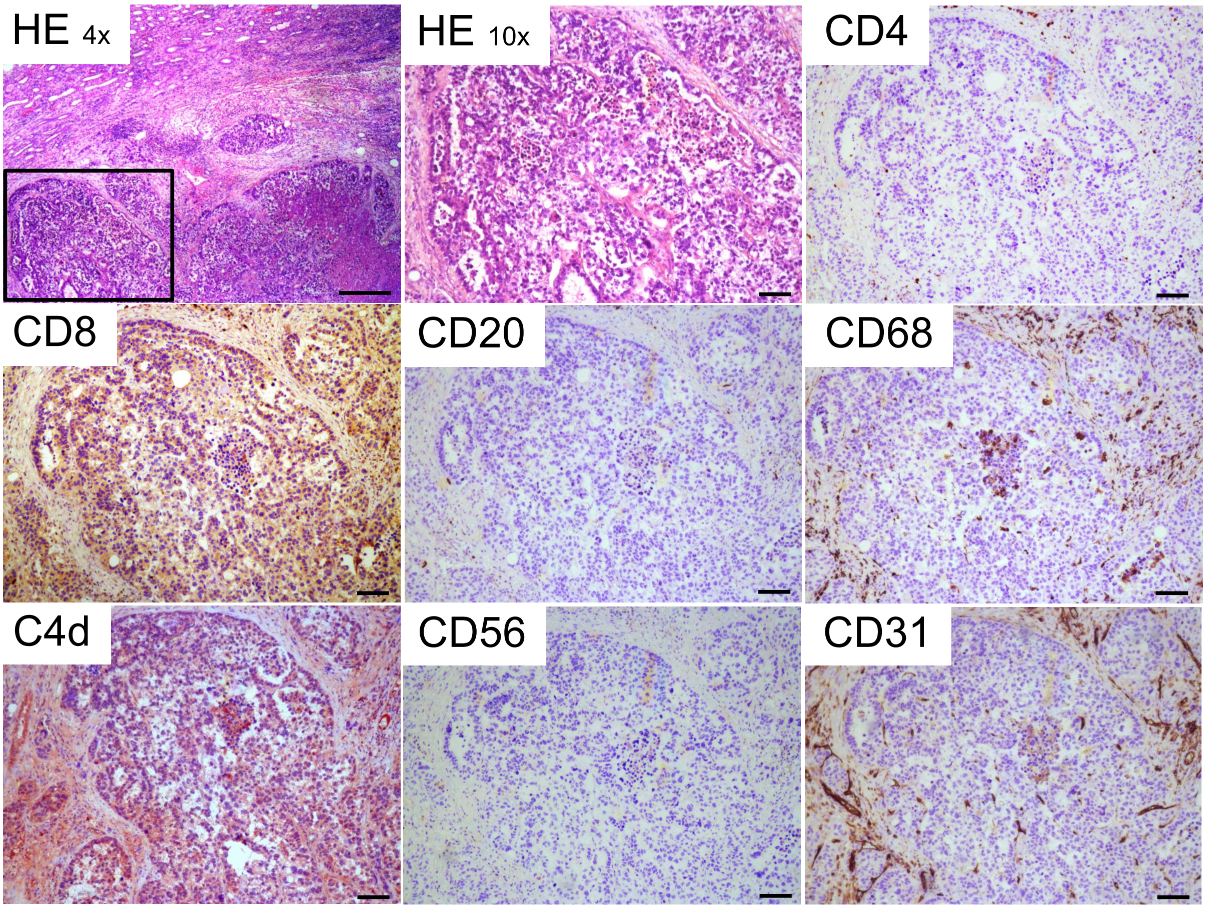

Figure S2: Histological sections showing the infiltration of the kidney by the tumor. 4x magnification hematoxylin and eosin staining (HE 4x). Scale bar: 400 µm. 10x magnification hematoxylin and eosin staining (HE 10x). Scale bar: 100 µm. CD4, CD8, CD20 (B lymphocyte), CD68 (macrophages), CD56 (NK cells), CD31 (blood vessels) cell staining and C4d staining (brown color).

References:

1. Bettens F, Buhler S, Tiercy JM. Allorecognition of HLA-C mismatches by CD8(+) T cells in hematopoietic stem cell transplantation is a complex interplay between mismatched peptide-binding region residues, HLA-C expression, and HLA-DPB1 disparities. *Front Immunol.* 2016;7:584.
2. Binggeli S, Egli A, Schaub S, et al. Polyomavirus BK-specific cellular immune response to VP1 and large T-antigen in kidney transplant recipients. *Am J Transplant.* 2007;7(5):1131–1139.
3. Leboeuf C, Wilk S, Achermann R, et al. BK polyomavirus-specific 9mer CD8 T cell responses correlate with clearance of BK viremia in kidney transplant recipients: first report from the Swiss Transplant Cohort Study. *Am J Transplant.* 2017;17(10):2591–2600.
